# Elbow instability secondary to lateral epicondylar nounion in an adult

**DOI:** 10.1007/s10195-012-0221-z

**Published:** 2012-12-11

**Authors:** Maria Valencia, Raul Barco, Samuel A. Antuña

**Affiliations:** Department of Orthopedic Surgery, Hospital La Paz, Paseo Castellana 261, 28046 Madrid, Spain

**Keywords:** Epicondylar nonunion, Varus elbow instability, Lateral collateral ligament complex

## Abstract

Symptomatic nonunion of the lateral epicondyle of the elbow is a rare injury. We present the case of a 36-year-old woman who complained of elbow pain and instability several months after a conservatively treated lateral epicondyle fracture that evolved into nonunion. In order to reestablish elbow stability, the patient underwent removal of the nonunited epicondylar fragment and ligament repair, with excellent clinical outcome.

## Introduction

Fractures of the lateral epicondyle are commonly seen in the pediatric population but are rare injuries in adults [1]. They occur more frequently as avulsion fractures during an episode of acute posterolateral or varus instability in which the lateral collateral ligament complex avulses a bone fragment with its attachment [2]. Nonunion after a conservatively treated lateral epicondylar fracture has been previously described in adults, but typically, these patients are asymptomatic without any complaint of elbow instability. We present the case of a 36-year-old woman with a symptomatic lateral epicondyle nonunion who underwent removal of and epicondylar fragment and ligament repair with an excellent clinical outcome.

## Case report

**Fig. 1:**
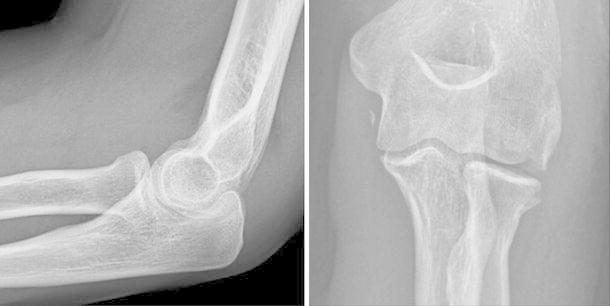
Anteroposterior and lateral radiographs of the injured elbow showing an avulsion fracture of the lateral epicondyle of the humerus

The patient gave informed consent to participate and was informed that data concerning the case would be submitted for publication. A 36-year-old right-hand-dominant woman sustained a fracture of the lateral epicondyle of her left elbow after a fall on the outstretched hand. The patient denied symptoms consistent with elbow dislocation or subluxation (Fig. 1). She was treated conservatively with 3 weeks’ immobilization in a cast. She was lost to follow-up, and 8 months later, she presented to our office with lateral elbow pain and a sensation of instability that were limiting her ordinary activities. Physical examination revealed pain to palpation over the lateral side of the elbow; range of motion was normal. When the elbow was compared with the uninjured side, there was increased varus laxity in extension and 30° of flexion, both in pronation and supination. The lateral pivot shift test was positive [2]. Neurovascular examination was normal.

**Fig. 2:**
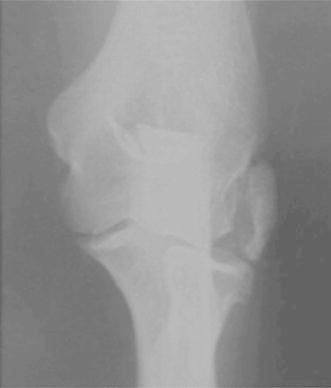
Radiographic examination 8 months after the original injury showing a nonunion of the epicondylar fragment extending distally and communicating with the joint

Radiographs of the elbow showed a displaced bony fragment compatible with nonunion of the lateral humeral epicondyle. There were also some very mild chronic osteoarthritic changes secondary to an old radial-head fracture and calcification in the area of the medial collateral ligament (Fig. 2).

**Fig. 3:**
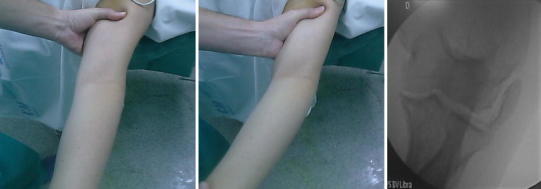
Varus stress to the elbow showed lateral instability, and fluoroscopy confirmed instability at the nonunion site

With the patient under general anesthesia, the elbow was examined under fluoroscopy and instability was confirmed, showing obvious instability at the nonunion site, with displacement of the epicondylar fragment when the elbow was stressed in varus (Fig. 3). Through a posterior skin incision, the lateral aspect of the elbow was approached. Once the nonunion site was identified, a sclerotic bone fragment that included the attachment of the extensor muscles and the lateral collateral ligament complex was isolated. A subperiosteal resection of the epicondyle was performed while trying to maintain as much ligament length as possible. After the sclerotic area of the distal humerus was refreshed until bleeding, the lateral ligament complex was reattached at the point of isometry with a transosseous suture through bone tunnels. The repair was augmented with a suture anchor and a running nonabsorbable suture within the ligament and capsule. At the end of the procedure, the elbow was checked for stability and motion. A standard closure was performed, and the limb was placed into a long-arm splint with the elbow flexed 90° and the forearm pronated. The patient was kept in the splint for 3 weeks, after which she started a 3-month active range of motion exercise and rehabilitation program.

**Fig. 4:**
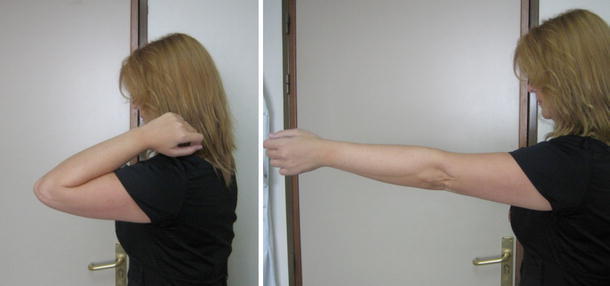
At final follow-up, 2 years after surgery, the patient was asymptomatic with full range of motion

**Fig. 5:**
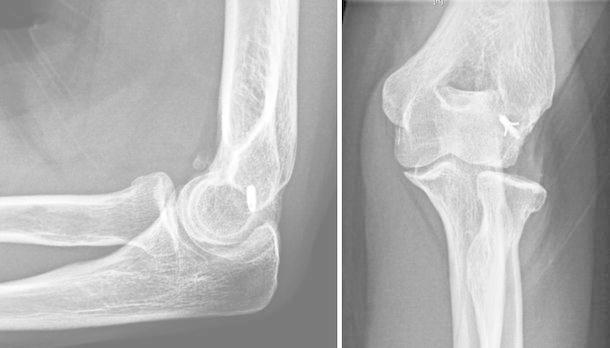
Radiographic examination after lateral epicondylectomy and reattachment of the lateral ligament complex with transosseous fixation and suture anchors

At final follow-up, 2 years afterward, the patient was free of pain and the feeling of instability was no longer present. On physical examination, the elbow had full range of motion and was stable (Fig. 4). The patient had a Mayo Elbow Performance Score of 100, had resumed her usual job, and denied any functional limitation in activities of daily living. Radiographs revealed no evidence of arthritic changes apart from sequelae of the radial-head fracture sustained 10 years earlier. (Fig. 5).

## Discussion

Avulsion fractures of the lateral humeral epicondyle in adults are rare. Although they can be caused by a direct blow to the elbow, they more frequently represent a bony avulsion of the lateral collateral ligament complex after a varus stress [3, 4]. The preferable treatment for these fractures remains controversial. Kobayashi et al. presented a series of 12 fractures of the lateral and medial epicondyles in adults [5]; they suggested that although surgical treatment provides good clinical results, conservative management could be selected for patients in whom the maximum diameter of the bone fragment is 13 mm or when displacement is <9 mm. Nonunion occurred in the majority of their patients treated conservatively, but none of them had any complaints or functional limitation. However, Gilchrist and McKee presented five adult patients with valgus instability of the elbow secondary to medial epicondyle nonunion [6]. To our knowledge, posterolateral elbow instability has not been previously described after an epicondylar nonunion. The reasons for an epicondylar nonunion becoming symptomatic are not known. It might be more frequent in throwing athletes and heavy laborers in whom the elbow is subjected to high-strain stresses at the fracture site [7]. However, it is probably more related to fragment size and extent of intra-articular involvement that may lead to communication of the fracture site with intra-articular fluid, which impairs bone healing. In this regard, we believe that examination under fluoroscopy may be helpful in identifying fractures with joint involvement and acute instability. The combination of intra-articular extent of the fracture and instability are probably the most determinant factors in nonunion development.

As this is a rare clinical scenario, the optimal treatment for established epicondylar nonunion with symptomatic instability remains unknown. Surgical treatment would be preferred if there is radiographic evidence of nonunion and the patient has significant functional impairment. Fracture fixation with or without bone grafting was been successfully used in children [8]. However, bone healing may be difficult to achieve in an adult due to small fragment size, limited contact area available with sclerotic avascular surfaces, and presence of a very high degree of strain. Excision of the epicondyle fragment and ligament advancement has been previously described for acute fractures and symptomatic medial epicondyle nonunion [6]. Excellent clinical results have been reported, obviating the need of bone grafting or a secondary procedure for hardware removal. Once the bone fragment is removed, the ligament is usually shortened to some degree, and it may be difficult to reattach it to its original position. Meticulous preservation of the entire available length and reattaching the ligament under the appropriate tension are essential. In our case, we combined a transosseous standard technique with a running suture to reinforce the repair.

In conclusion, lateral epicondyle fractures are rare injuries that merit careful evaluation in the acute setting. Large fragment size, evidence of instability, and fracture extension into the joint are probably the most predisposing factors for nonunion. If there is a symptomatic nonunion, fragment excision and ligament reattachment may lead to an excellent functional outcome.
